# Four new species of Cortinariaceae (Agaricales) from Northwestern China

**DOI:** 10.3389/fmicb.2024.1454736

**Published:** 2024-09-25

**Authors:** Longfei Fan, Xue Zhong, Tianfu Ma, Hongmin Zhou, Biyue Wang, Xiaohong Ji

**Affiliations:** ^1^College of Plant Protection, Gansu Agricultural University, Lanzhou, China; ^2^Ecology and Nature Conservation Institute, Chinese Academy of Forestry, Beijing, China; ^3^College of Biodiversity Conservation, Southwest Forestry University, Kunming, China; ^4^College of Pharmacy and Life Sciences, Jiu Jiang University, Jiujiang, China

**Keywords:** *Cortinarius*, ITS + LSU, morphology, phylogeny, taxonomy

## Abstract

**Introduction:**

Cortinariaceae, which belongs to the Agaricales order, is a globally recognized family, known for its high species diversity.

**Methods:**

Eight internal transcribed spacer (ITS) and nuclear large ribosomal subunit (LSU) sequences were newly generated, and phylogenetic analyses were performed by combining ITS and LSU datasets. Four species were identified as forming four independent lineages with robust support in phylogenies based on both datasets.

**Results:**

These new species in the taxa, *Cortinarius gansuensis*, *Cortinarius tricholomoidus*, *Cortinarius vinoso-griseum*, and *Phlegmacium subcalyptratum* from Northwestern China are described and illustrated based on morphological and molecular evidence. *Cortinarius gansuensis* is characterized by a convex and brownish vinaceous pileus, generative hyphae with clamp connections, and ellipsoid basidiospores (8.5–10.6 μm × 5.4–6.8 μm); *Cortinarius tricholomoidus* is characterized by a broadly umbonate and snuff brown pileus, generative hyphae with clamp connections, and broadly ellipsoid to subglobose basidiospores (7.4–8.5 μm × 6.2–7.3 μm); *Cortinarius vinoso-griseum* is characterized by a violaceous gray pileus, generative hyphae with clamp connections, and smaller basidiospores (7.5–9.7 μm × 5.6–7.8 μm); and *Phlegmacium subcalyptratum* is characterized by a small and apricot-orange pileus, generative hyphae with clamp connections, and fusiform basidiospores (10.0–12.7 μm × 5.6–6.8 μm).

**Discussion:**

Full descriptions, illustrations, and results of phylogenetic analyses of the four species along with discussions on related species are provided.

## 1 Introduction

The species within the family Cortinariaceae are crucial ectomycorrhizal fungi associated with various plants, including the gymnosperms, rosid angiosperms, and some shrubs. The genus is typically characterized by mostly brown and dark-spored basidiospores (Matheny et al., 2015; Dentinger et al., 2016).

The family Cortinariaceae includes 10 genera, *Aureonarius* Niskanen & Liimat., *Austro-cortinarius* Niskanen & Liimat., *Calonarius* Niskanen & Liimat., *Cortinarius* (Pers.) Gray, *Cystinarius* Niskanen & Liimat., *Hygronarius* Niskanen & Liimat., *Mystinarius* Niskanen & Liimat., *Phlegmacium* (Fr.) Wünsche, *Thaxterogaster* Singer, and *Volvanarius* Niskanen & Liimat. (Liimatainen et al., 2022). Among these genera, *Cortinarius* is the largest genus in the Agaricales with a worldwide distribution. However, the taxonomic status of this genus has long been a subject of controversy owing to the overlapping morphological characteristics. Numerous sections or clades have been proposed to address this issue (Brandrud, 1998; Niskanen et al., 2016; Soop et al., 2019; Zhang et al., 2023; Long et al., 2024). The currently recognized subgenera include *Cortinarius*, *Camphorati*, *Dermocybe*, *Illumini*, *Infracti*, *Iodolentes*, *Leprocybe*, *Myxacium*, *Orellani*, *Paramyxacium*, and *Telamonia* (Liimatainen et al., 2022). *Cortinarius* s.l. is a cosmopolitan genus with 313 accepted taxa, including 23 taxa originally described from Northwestern China. In addition, there are at least over 5,000 accepted species in *Cortinarius* s.s.

The species of *Leprocybe* are distributed in both the Northern and Southern Hemispheres, and this subgenus is characterized by small-to medium-sized basidiomes, mostly agaricoid or sequestrate. A morpho-genetic revision of the Northern Hemispheric *Leprocybe* was conducted by Ammirati et al. (2021) and Bidaud et al. (2021). Currently, seven sections are included in the genus *Leprocybe*: *Leprocybe*, *Fuscotomentosi*, *Melanoti*, *Persplendidi*, *Squamiveneti*, *Veneti*, and *Veronicae*.

*Phlegmacium* (Fr.) (Liimatainen et al., 2022), distributed in the Northern Hemisphere, is characterized by medium-to large-sized, predominantly stipitocarpic, yellow agaricoid (phlegmacioid) basidiomes. Four subgenera were recognized: *Phlegmacium*, *Bulbopodium*, *Carbonella*, and *Cyanicium*.

In this study, we focused on Cortinariaceae represented by eight specimens from Gansu, China. Phylogenetic analyses based on the internal transcribed spacer (ITS) and nuclear large ribosomal subunit (LSU) rDNA sequences were carried out, and four new species were recognized. The current study aimed to further explore the species diversity of Cortinariaceae in the Gansu province of Northwestern China and confirm the taxonomic position of *Phlegmacium* within the Cortinariaceae family.

## 2 Materials and methods

### 2.1 Morphological studies

All specimens were deposited in the Fungal Herbarium of Gansu Agricultural University (MHGAU, China) and the National Institute of Occupational Health and Poison Control, Chinese Center for Disease Control (NIOHP, China CDC). The size of the basidiomes, as determined by the pileus width, was described as small (<5.0 cm), medium (5.0–9.0 cm), and large (> 9.0 cm). The samples were recorded in the growth environment and geographical location and photographed while fresh; then, they were dried using a food dehydrator (30–35°C for several hours till dry) and stored in a refrigerator (−20°C) for morphological studies. The morphological descriptions were based on field notes and dried specimens. The microscopic features were examined and described in 5% KOH, 1% Phloxine B (C_20_H_4_Br_4_Cl_2_K_2_O_5_), or Melzer’s reagent and observed under a Nikon Eclipse 80i microscope (Nikon, Tokyo, Japan) with a magnification of up to ×1,000 following Fan et al. (2021) and Long et al. (2024). Thirty basidiospores were measured per collection (excluding apiculus and ornamentation), and the averages (av. X) and quotients (av. Q = L/B) were calculated. L = mean spore length, B = mean spore width, and Q = variation in the ratios of L/B between the specimens studied. Color terms were cited from Royal Botanic Garden Edinburgh (1969) and Kornerup and Wanscher (1978).

### 2.2 Molecular phylogeny

A Phire® Plant Direct PCR Kit was used to obtain PCR products from the dried specimens according to the manufacturer’s instructions and as described previously by Long et al. (2024), with some modifications. The primer pairs ITS5 and ITS4 for ITS and LR0R and LR7 for nrLSU were used to amplify the internal transcribed spacer (ITS) and large ribosomal subunit (LSU), respectively (White et al., 1990). The PCR procedure for the ITS was as follows: initial denaturation at 98°C for 5 min, followed by 35 cycles at 94°C for 5 s, 54°C for 5 s, and 72°C for 5 s, and a final extension of 72°C for 10 min. The PCR procedure for the nrLSU was as follows: initial denaturation at 94°C for 1 min, followed by 35 cycles at 94°C for 30 s, 50°C for 1 min, and 72°C for 1.5 min, and a final extension of 72°C for 10 min (Fan et al., 2021). The PCR products were purified and sequenced by Engine Biotech, China. The newly generated sequences from this study have been deposited in GenBank and are listed in Table 1.

**Table 1:** Information on the sequences used in this study.

Species	Sample no.	Country	GenBank	
			ITS	nLSU
*Cortinarius anomalus*	CFP1154 typus	Sweden	KX302224	
*C. anomalus*	CA3	Norway	KC842425	KC842495
*C. anomalus*	TUB011883	Europe, Germany	AY669645	AY669645
*C. barlowensis*	JFA13140	North America	FJ717554	
*C. bolaris*	T40	Europe, Norway	KC842426	KC842496
*C. bolaris*	TUB0118524	Germany	AY669596	AY669596
*C. bolaris*	3,861	Canada	KJ705110	
*C. bolaris*	CFP1008 typus	Sweden	KX302233	
*C. calaisopus*	PDD103678	New Zealand	KF727395	KF727338
*C. calaisopus*	PDD94050	Dunedin, New Zealand	NR157880	MH108373
*C. camphoratus*	SMI193	Canada, North America	FJ039626	
*C. camphoratus*	TRTC175623	Canada	PP383785	
*C. caninus*	HMJAU44372	China	OP620657	OP620671
*C. caninus*	CFP627 typus	Sweden	KX302250	
*C. cinnamomeus*	UBCF19609	Canada	HQ604650	HQ604650
*C. cinnamomeus*	OS480	Norway	KC842413	KC842483
*C. cotoneus*	19XML11153	China	OP620655	OP620666
*C. cotoneus*	OS579	Norway	KC842423	KC842493
*C. cruentoides*	PDD101864 typus	New Zealand	KJ635217	KJ635217
*C. cruentoides*	JAC13529	New Zealand	MW263695	MW263408
*C. delibutus*	F17048	Canada, North America	FJ717515	
*C. delibutus*	OS574	United States, North America	KC842441	KC842511
*C. dysodes*	PDD70499 typus	New Zealand	GU233340	GU233394
*C. dysodes*	PDD72664	New Zealand	MH101614	MH108334
*C. epsomiensis*	KM74963 typus	United Kingdom	MK010952	
*C. epsomiensis*	HMJAU44505	China	ON254423	
*C. ferrugineifolius*	IBMMoser19910305	Europe, North America	NR171327	
*C. ferrugineifolius*	SHLindstromCFP969	Europe, North America	MT935278	
*C. ferrusinus*	JB810613	Spain	KY657254	
*C. ferrusinus*	JB888116	Spain	KY657255	
*C. fibrillososalor*	MHHNU32070	East Asia, China, Hunan	OR660685	OR647503
*C. fibrillososalor*	MHHNU32494	East Asia, China, and Hunan	OR647481	OR647506
*C. flammeouraceus*	H6029919	Europe, North America	NR170035	
*C. flammeouraceus*	HMJAU60648	China	OL891470	
*C. fusisporus*	BILAS51600	Lithuania	ON406294	
*C. fusisporus*	BILAS51540	Lithuania	ON261481	
***C. gansuensis***	**FLF814**	**China**	**PP911501**	**PP907035**
***C. gansuensis***	**WBY814**	**China**	**PP911502**	**PP907036**
*C. illibatus*	HMJAU48760	China	MW911735	OP620668
*C. illibatus*	iNat13972929	United States	OK346478	
*C. indotatus*	PDD88257	New Zealand	KJ421110	KJ421110
*C. indotatus*	PDD92040	New Zealand	GU222322	
*C. liyui*	HMJAU58939 typus	Jilin, China	OP620660	OP620672
*C. liyui*	HMJAU58938	Jilin, China	OP620661	
*C. luhmannii*	TUB019811	Germany	KJ421114	
*C. luhmannii*	TUB019808	Germany	KJ421111	
*C. pseudocamphoratus*	HMJAU48698 holotype	China	OM001483	OM001524
*C. pseudocamphoratus*	HMJAU48798	China	OM001489	
*C. pseudosalor*	MHHNU8349	East Asia, China, and Hunan	OR647352	
*C. pseudosalor*	MHHNU32148	East Asia, China, and Hubiei	OR660688	OR647505
*C. pseudosalor*	MHHNU32082	East Asia, China, and Hubiei	OR660686	OR647504
*C. putorius*	TNO7411HT	United States, North America	KR011124	
*C. rotundisporus*	PDD96298	New Zealand	MH101550	MH108389
*C. rotundisporus*	PDD72611	Australia, New Zealand	AY669612	AY669612
*C. salor*	TUB011838	Europe, Germany	AY669592	AY669592
*C. sommerfeltii*	HMJAU44457	China	OP620652	OP620663
*C. sommerfeltii*	SOMF30854	Spain	OQ398585	
*C.* sp.	SWUBC741	Canada	DQ481671	
*C.* sp.	T21468	China	OP620656	OP620667
*C.* sp.	MEL2089705	Australia	GQ890326	JX544951
*C. spilomeus*	TUB011523	Europe	AY669654	AY669654
*C. spilomeus*	CFP1137 typus	Sweden	KX302267	
*C. spilomeus*	H6031514	Finland	KX302264	
*C. subargyronotus*	H7018127	Finland	NR131871	
*C. subargyronotus*	C358	Hungary	OP099768	
*C. subsalor*	HMJAU48759 typus	China	MW911734	OP620670
*C. subsalor*	HMJAU48758	China	MW911733	
*C. subsanguineus*	HMJAU48961	China	OP620653	OP620664
*C. subsanguineus*	HMAS250503	China	MK411450	
*C. subtortus*	F16111	North America	FJ157044	FJ157044
*C. subtortus*	TUB011382	Europe	AY174857	AY174857
*C. subtropicus*	MHHNU31981	East Asia, China, and Hunan	OR660687	OR647502
*C. subtropicus*	MHHNU33533	East Asia, China, and Hunan	OR647488	OR647508
*C. tabularis*	CFP949 typus	Sweden	KX302275	
*C. tabularis*	H7022440	Finland	KX302279	
*C. tasmacamphoratus*	HOA20606A0	Tasmania	AY669633	AY669633
*C. tessiae*	PDD107517	New Zealand	MG019356	MG019356
*C. tetonensis*	JFA10350	North America	MZ580436	
*C. tibeticisalor*	HMJAU48764 typus	China	MW911729	OP620669
*C. tibeticisalor*	HMJAU48763	China	MW911730	
***C. tricholomoidus***	**FLF806**	**China**	**PP911497**	**PP907031**
***C. tricholomoidus***	**FLF827**	**China**	**PP911498**	**PP907032**
*C. uliginosus*	KH7	Norway	KC842412	KC842482
*C. uliginosus*	TUB011823	Germany	AY669584	KJ403804
*C. umbrinolens*	TUB011918	Germany	AY669658	
*C. umbrinolens*	NFSG20231021	Britain	PP355760	
*C. veronicae*	PDD68468 typus	New Zealand	KC017355	
*C. veronicae*	JAC10781	New Zealand	MW263653	MW263361
***C. vinoso-griseum***	**FLF463**	**China**	**PP911499**	**PP907033**
***C. vinoso-griseum***	**WBY463**	**China**	**PP911500**	**PP907034**
*C. viridipileatus*	OTA61977	New Zealand	MK546592	MK546595
*C. viridipileatus*	OTA64087	New Zealand	MK546593	MK546596
*C. xiaojinensis*	HMJAU58895	China	OP620654	OP620665
*C. xiaojinensis*	HMAS274355	China	MK411447	
*C. saginus*	T30	Norway	KC842448	KC842518
*C. saginus*	IB19960705	United States	AF325608	AF388768
*Phlegmacium caerulescens*	Fungal	Turkish	MH718791	MH718792
*P. caerulescens*	SF44815	Frisia	NR130199	
*P. calyptratus*	iNAT18441433	America	OL602058	
*P. calyptratum*	MICH10328	Frisia	NR130201	
*P. glaucocephalus*	IB19950679	Frisia	NR130221	
*P. glaucocephalus*	HBAU15487	China	MW862302	
*P. neotriumphans*	G2970631	Frisia	NR157947	
*P. neotriumphans*	HMAS260251	China	OK490097	
*P. populinum*	OF58605	Norway	MT216235	
*P. populinum*	O58647	Europe, Australia, Tasmania, New Zealand, and South America	AY669521	
***P. subcalyptratum***	**FLF849**	**China**	**PP911503**	**PP907037**
***P. subcalyptratum***	**WBY849**	**China**	**PP911504**	**PP907038**
*C. eartoxicus* (outgroup)	MEL2351137	Australia	KP311432	KP311376
*C. eartoxicus* (outgroup)	MEL2151441	Australia	OK159884	
*C. orellanus* (outgroup)	IB19980580	Austria	AF389164	AF388773
*C. rubellus* (outgroup)	TUB011828	Germany	AY669595	AY669595

The samples used in this study are in bold.

The new sequences generated and additional sequences retrieved from GenBank (Table 1) were aligned using BioEdit 7.0.5.3 and ClustalX 1.83, followed by manual adjustments. The sequences of the Orellana clade including *Cortinarius rubellus* Cooke, *C. orellanus* Fr., and *C. eartoxicus* Gasparini were utilized as outgroups (Dima et al., 2021). The phylogenetic analysis was conducted based on the maximum parsimony (MP), maximum likelihood (ML), and Bayesian inference (BI) methods. The best-fit model was selected using ModelFinder (Kalyaanamoorthy et al., 2017), with the Akaike information criterion (AIC) guiding the process.

The MP, ML, and BI analyses were performed using PAUP on XSEDE (4.a165), RAxML 8.2.12, and MrBayes 3.2.5, respectively (Ronquist et al., 2012). Four Markov chains were run for two independent runs from random starting trees for 10 million generations, with the trees sampled every 1,000 generations. The burn-in was set at 25% to discard initial trees. The sequence alignment was deposited in TreeBase (submission ID: 31486). The branches with bootstrap support for the MP, ML (BP), and Bayesian posterior probabilities (BPPs) greater than or equal to 50% (BP) and 0.90 (BPP) were considered significantly supported.

## 3 Results

The ITS+LSU dataset comprised 112 fungal collections representing approximately 65 taxa of the genus *Cortinarius*. ModelFinder suggested that GTR + I + G was the best-fit model of nucleotide evolution for the BI. The Bayesian analysis resulted in a concordant topology with an average standard deviation of split frequencies of 0.000067. The MP, ML, and BI analyses resulted in nearly identical topologies, and thus, only the ML tree was presented with the bootstrap supports for BP and BPP when they were greater than or equal to 50% and 0.90, respectively.

**Figure 1:**
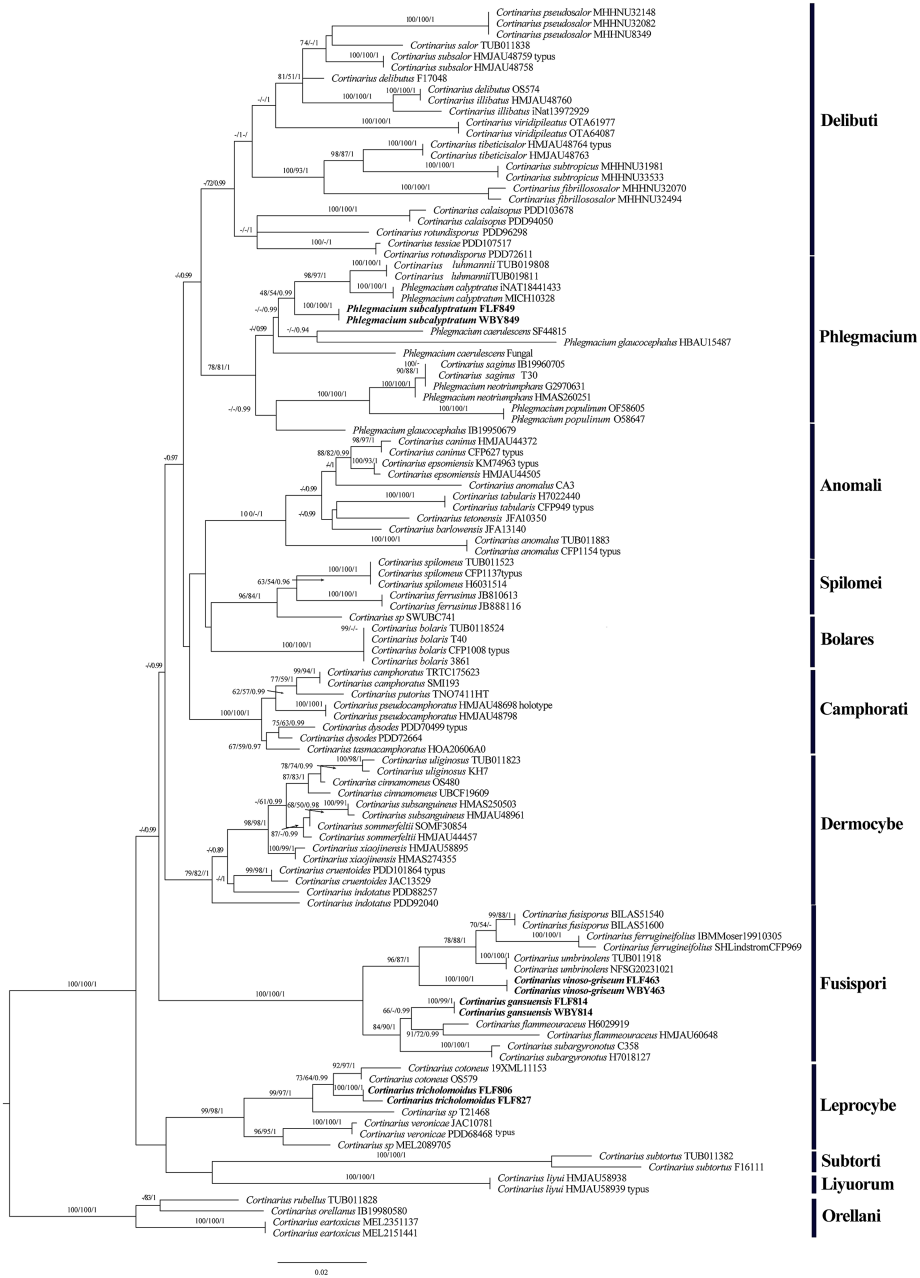
Phylogeny of *Cortinarius* s. l. by ML analysis based on the ITS + LSU dataset. The branches are labeled with parsimony bootstrap proportions, maximum likelihood bootstrap >50%, and Bayesian posterior probabilities >0.90. The new species are in bold.

Our phylogeny, which was inferred from the ITS+LSU sequences (Figure 1), was similar to the research by Zhang et al. (2023) and Long et al. (2024). The phylogenetic analysis showed 12 sections, sect. *Delibuti*, *Phlegmacium*, *Anomali*, *Spilomei*, *Bolares*, *Camphorati*, *Dermocybe*, *Fusispori*, *Leprocybe*, *Subtorti*, *Liyuorum*, and *Orellani*, and each section formed separate monophyletic lineages with strong statistical support.

The section *Fusispori* formed a distinct, high-supported clade (BP = 100 and BPP = 1) and was separated from other sections. Two new species, namely *Cortinarius gansuensis* and *Cortinarius vinoso-griseum*, nested within the sect. *Fusispori* clade. *Cortinarius tricholomoidus*, nested within the sect. *Leprocybe* clade, formed an independent lineage with high statistical support (100/100/1). It is worth noting that the collections of *Phlegmacium subcalyptratum* were nested within the sect. *Phlegmacium* clade (Figure 1).

## 4 Taxonomy

**Figure 2:**
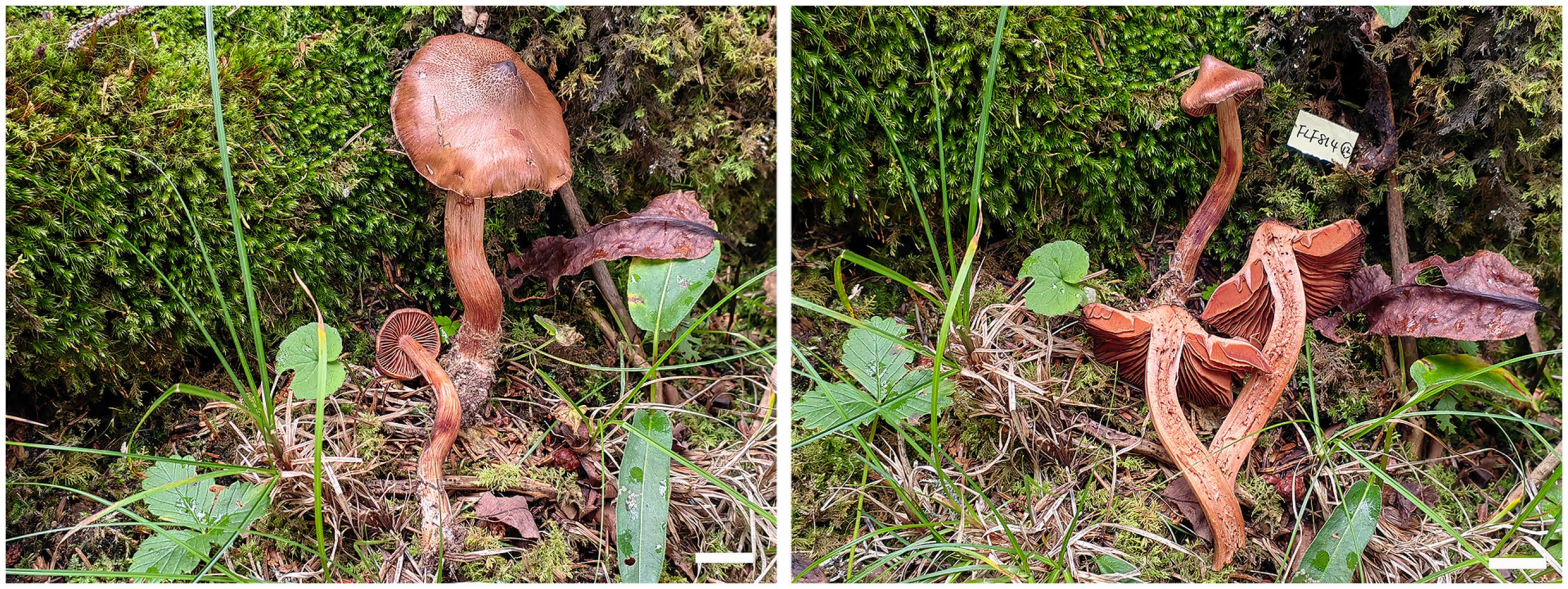
Basidiomes of *Cortinarius gansuensis* (Holotype, FLF 814). Bars: 1 cm.

**Figure 3:**
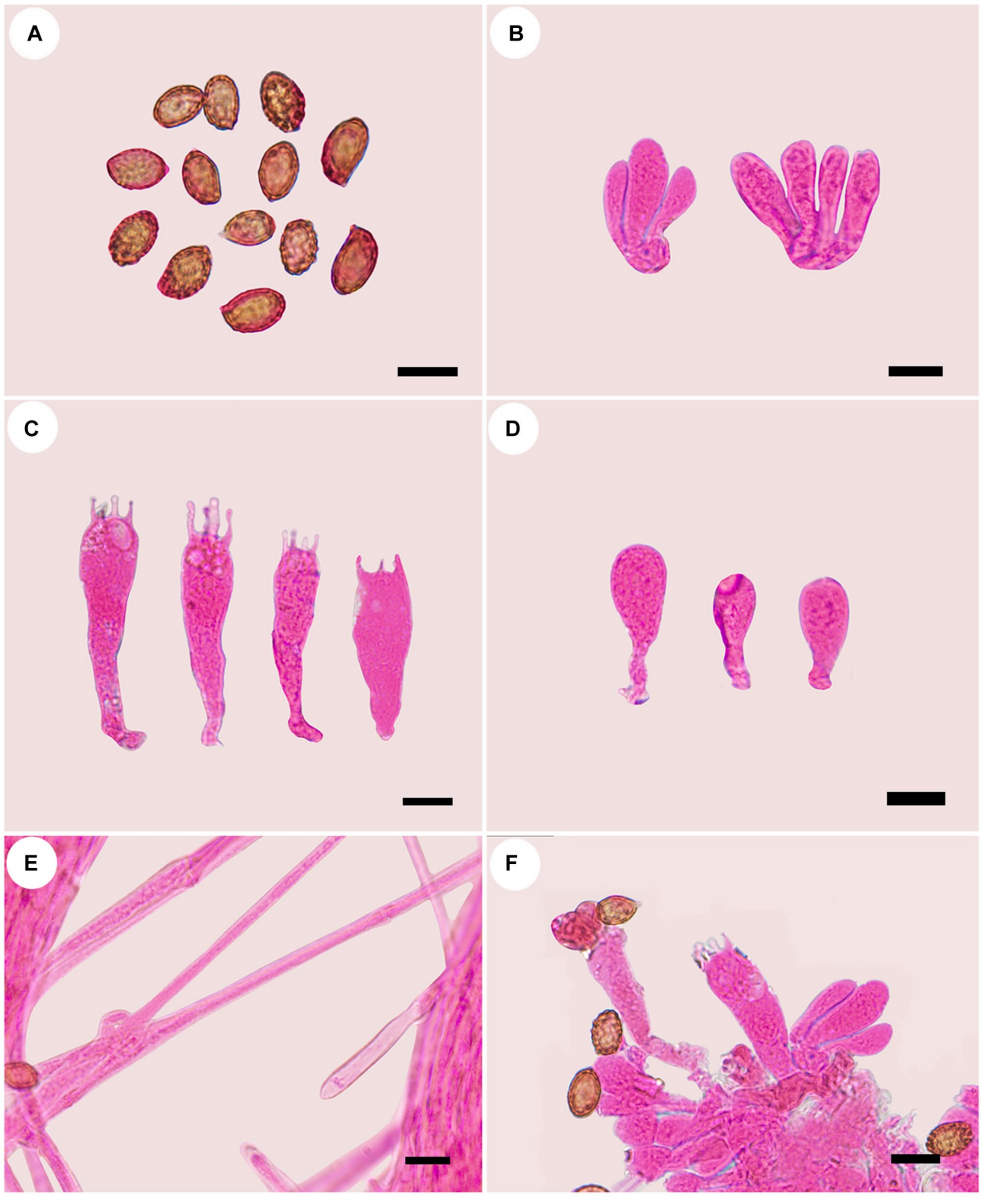
Microscopic structures of *Cortinarius gansuensis* (holotype, FLF 814). **(A)** Basidiospores; **(B)** Basidioles; **(C)** Basidia; **(D)** Cystidia; **(E)** Hyphae with clamp connections; **(F)** A section of the hymenium. Scales: 10 μm.

***Cortinarius gansuensis* B.Y. Wang, T.F. Ma & L.F. Fan, sp. nov.** (Figures 2, 3).

**MycoBank No**: 854910.

**Etymology**: Gansuensis (Latin) refers to the species found in Gansu Province.

**Holotype**: CHINA. Gansu Province, Zhuoni County, Dayu Valley, July 2023, FLF 814 (MHGAU).

**Diagnosis**: Differs from *C*. *flammeouraceus* by having longer basidiospores.

**Description**: Basidiomes small-to medium-sized. Pileus 1.6–3.6 cm, conical to hemispherical, convex to plane with an umbo, broadly umbonate at the center, margin incurved; at first brownish vinaceous, tinged darker brown at the center, with brown universal veil remains at the margin; surface silky when dry or glutinous when wet. Context thin, brown, soft. Lamellae adnate to adnexed, lilac, moderately distant, sometimes margin wavy. Stipe cylindrical to clavate, 3.8–4.5 cm long, 4.0–6.0 mm wide, brown, leaving a brownish ring on the upper stem, hollow. Odor indistinct.

Basidiospores [100/5/5] (8.1–)8.5–10.6(−11.3) × 5.4–6.8(−7.2) μm, av. 9.6 × 6.1 μm, Q = 1.6, ellipsoid, yellowish brown, moderately verrucose, without amyloid and dextrinoid reaction. Basidia (28.0–)45.8–49.6 × (8.2–)8.6–10.7 μm, 4-spored, sterigmata up to 4.0 μm, clavate to subcylindrical, colorless or with granules. Pileipellis duplex, hyphae 58.0 μm wide, epicutis gelatinous, 35.0–50.0 μm thick, composed of colorless or amber yellow, irregularly arranged and strongly interwoven hyphae, hypocuits 25.0–40.0 μm thick, composed of colorless or amber yellow, nearly parallel cylindrical hyphae. Lamellar edges fertile. Cystidia absent. Lamellar trama regular, 40.0–80.0 μm thick, composed of parallel arranged hyphae, hyphae 4.0–8.0 μm wide, with clamp connections. Stipitipellis gelatinous, stipe hyphae 9.0–11.0 μm wide, thin-walled, cylindrical, interwoven.

**Habitat, ecology, and distribution**: Solitary on mixed coniferous and broad-leaved forestland, known from Gansu, China, July to September. Additional specimens examined. China, Gansu Province: Zhuoni County, Taohe National Nature Reserve, at 29.769154°N, 110.086577°E, alt. 1,405 m, 14 September 2023, L.F. Fan and B.Y. Wang, (WBY 814, MHGAU).

**Notes**: *Cortinarius gansuensis* can be differentiated from other species of section *Fusisori* for its conical pileus, usually under mixed coniferous and broad-leaved forestland at 1,405–1,500 m. In addition, basidiospores ellipsoid, rarely subglobose, while other members in this section usually subglobose to broadly ellipsoid.

**Figure 4:**
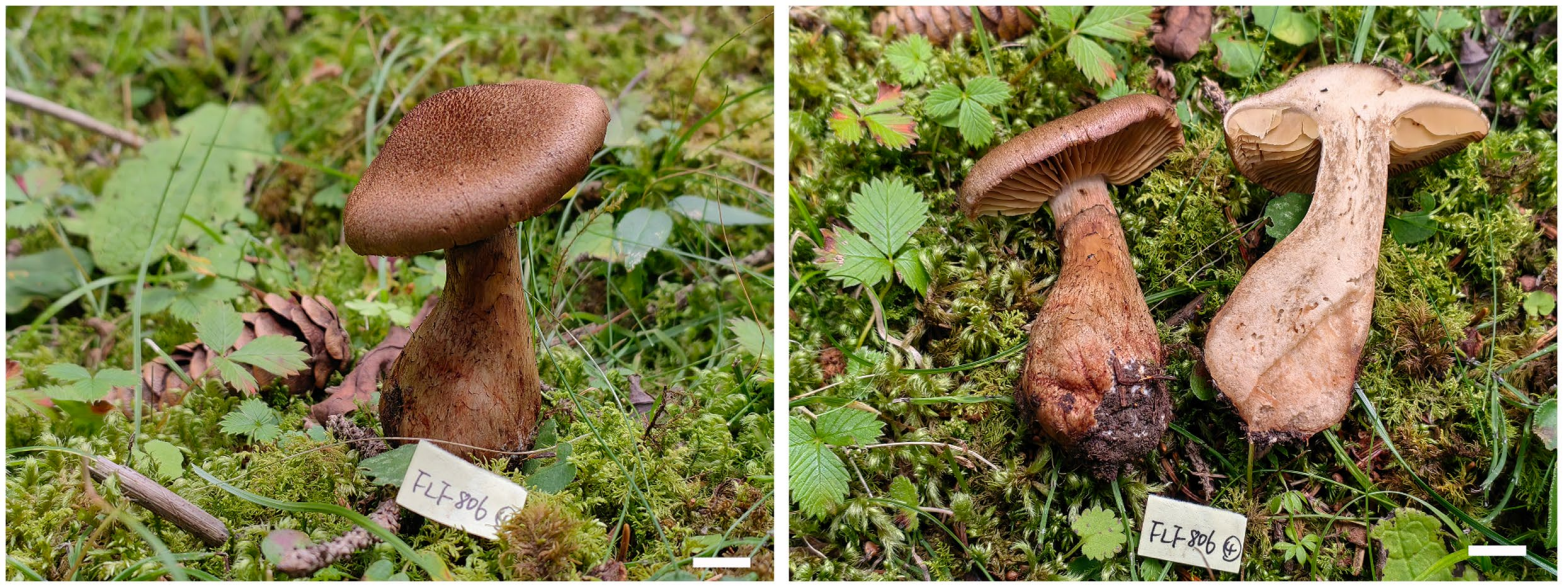
Basidiomes of *Cortinarius tricholomoidus* (Holotype, FLF 806). Bars: 1 cm.

**Figure 5:**
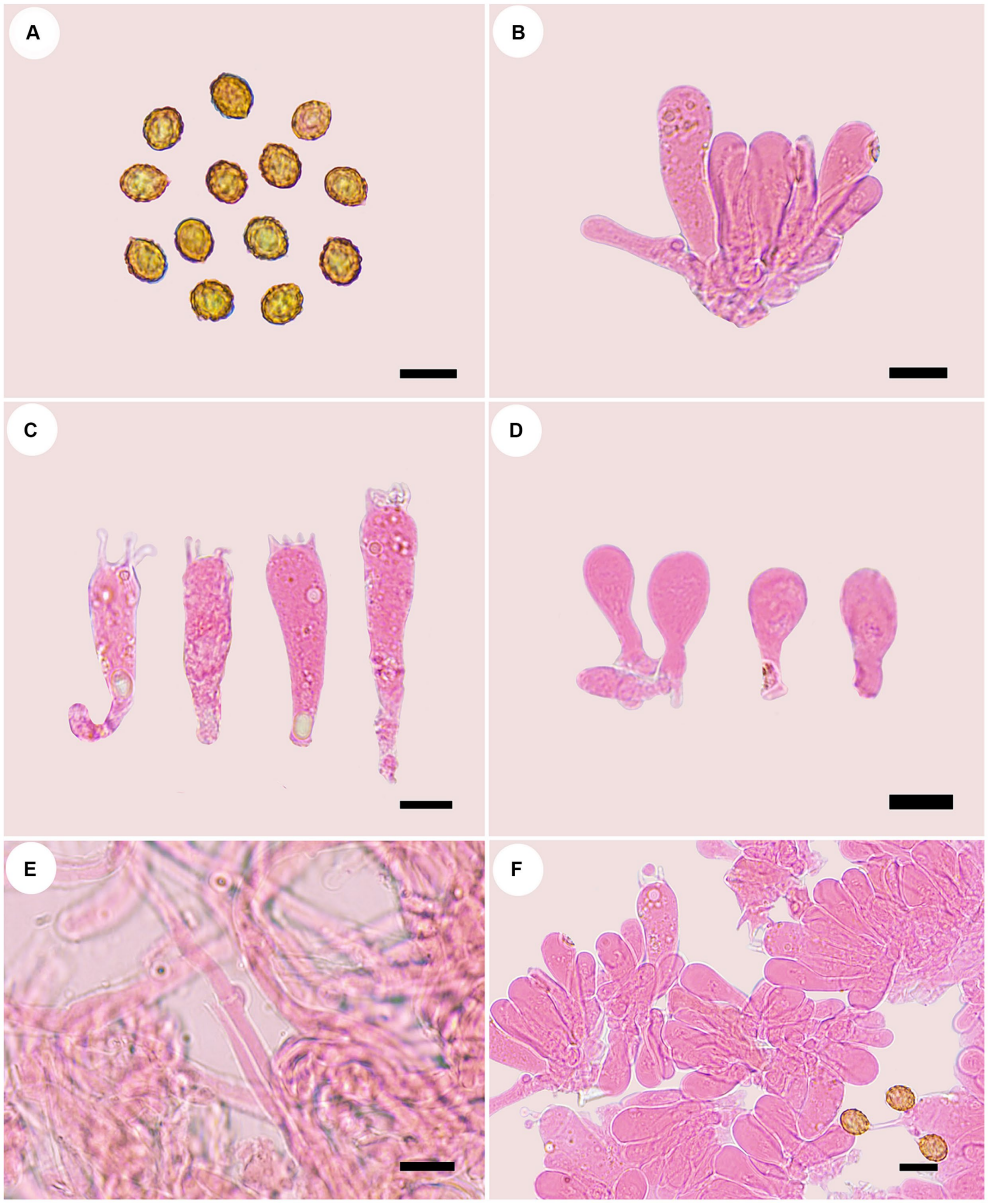
Microscopic structures of *Cortinarius tricholomoidus* (holotype, FLF 806). **(A)** Basidiospores; **(B)** Basidioles; **(C)** Basidia; **(D)** Cystidia; **(E)** Hyphae with clamp connections; **(F)** A section of the hymenium. Scales 10 μm.

***Cortinarius tricholomoidus* B.Y. Wang, T.F. Ma & L.F. Fan, sp. nov.** (Figures 4, 5).

**MycoBank No**: 854911.

**Etymology**: Tricholomoidus (Latin) refers to the stipe or the shape of this species being similar to *Tricholoma* spp.

**Holotype**: CHINA. Gansu Province, Zhuoni County, Boyu Valley, September 2023, FLF 806 (MHGAU).

**Diagnosis**: Differs from the *C. gansuensis* by its shorter stipe.

**Description**: Basidiomes small-to medium-sized. Pileus 3.0–6.0 cm, broadly umbonate at the center, margin incurved; at first snuff brown, tinged umber at the center, with brown universal veil remains at margin; surface finely felty. Context brown, soft. Lamellae adnate to adnexed, lilac, moderately distant. Stipe cylindrical to clavate, 6.1–7.6 cm long, 1.3–1.6 mm wide, brown, leaving a brownish ring on the upper stem, hollow. Odor indistinct.

Basidiospores [100/5/5] (7.1–)7.4–8.5(−8.7) × (5.9–)6.2–7.3(−7.4) μm, av. 7.98 × 6.78 μm, Q = 1.18, broadly ellipsoid to subglobose, yellowish brown, moderately verrucose, without amyloid and dextrinoid reaction. Basidia 32.0–47.0 μm × 9.0–11.0 μm, four-spored, sterigmata up to 4.0 μm, clavate to subcylindrical, colorless or with granules. Pileipellis duplex, hyphae 4.0–8.0 μm wide, epicutis strongly gelatinous, 88.0–120.0 μm thick, composed of colorless or amber yellow, irregularly arranged and strongly interwoven hyphae, hypocuits 20.0–30.0 μm thick, composed of colorless or amber yellow, nearly parallel cylindrical hyphae. Lamellar edges fertile. Cystidia absent. Lamellar trama regular, 50.0–60.0 μm thick, composed of parallel arranged hyphae, hyphae 4.0–8.0 μm wide, with clamp connections. Stipitipellis gelatinous, stipe hyphae 3.0–6.0 μm wide, thin-walled, cylindrical, interwoven.

**Habitat, ecology and distribution**: Usually solitary on coniferous forestland, from Gansu, China, July to September. Additional specimens examined. China, Gansu Province: Zhuoni County, Taohe National Nature Reserve, at 34.563761°N, 103.553064°E, alt. 2,845 m, 13 September 2023, L.F. Fan and B.Y. Wang, (FLF 827, MHGAU).

**Notes**: *Cortinarius tricholomoidus* can be differentiated from other species of section *Leprocybe* for its finely felty pileus, usually under coniferous forestland at 2,845 m. In addition, basidiospores broadly globose to subglobose.

**Figure 6:**
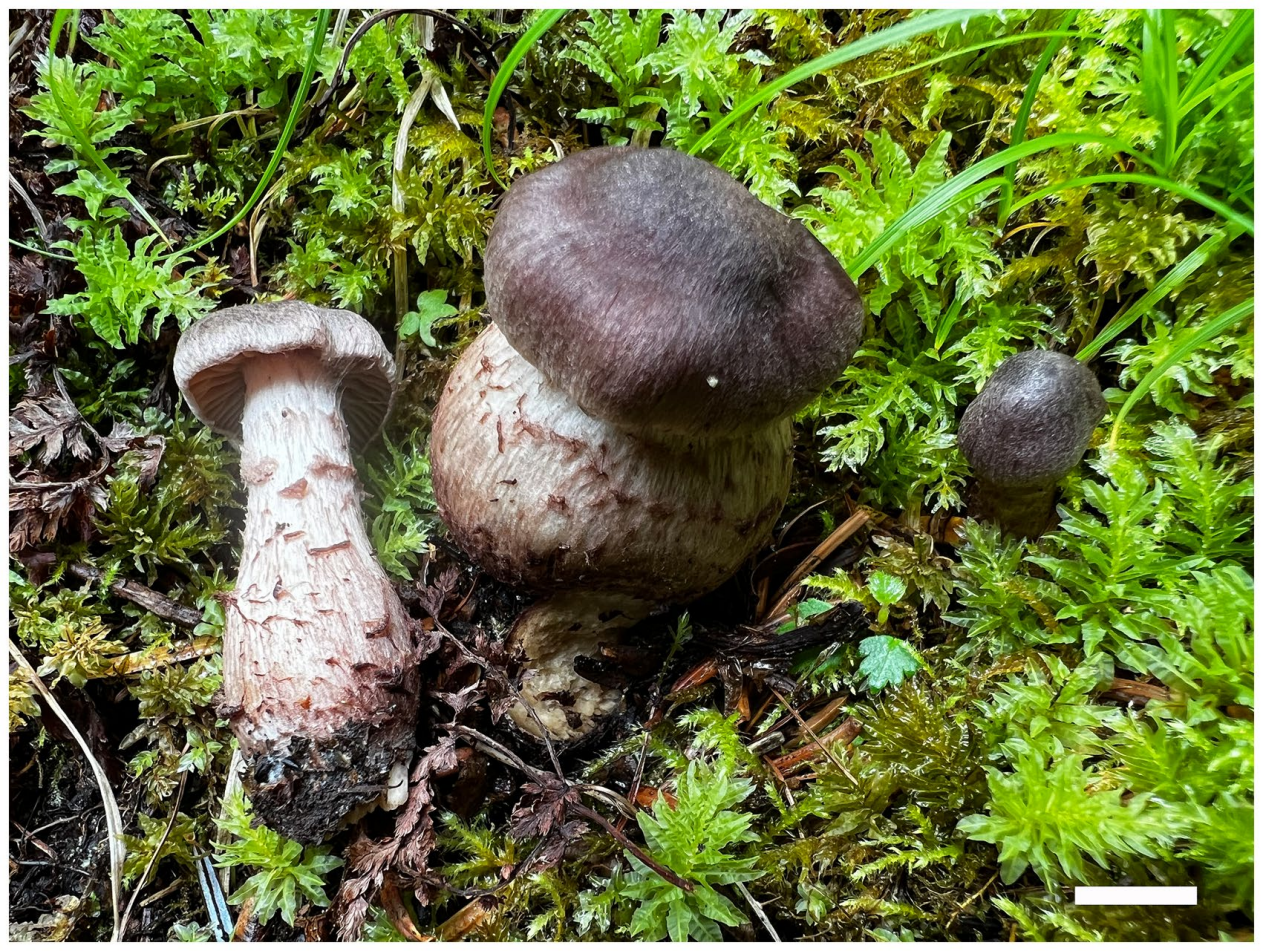
Basidiomes of *Cortinarius vinoso-griseum* (Holotype, FLF 463). Bar: 1 cm.

**Figure 7:**
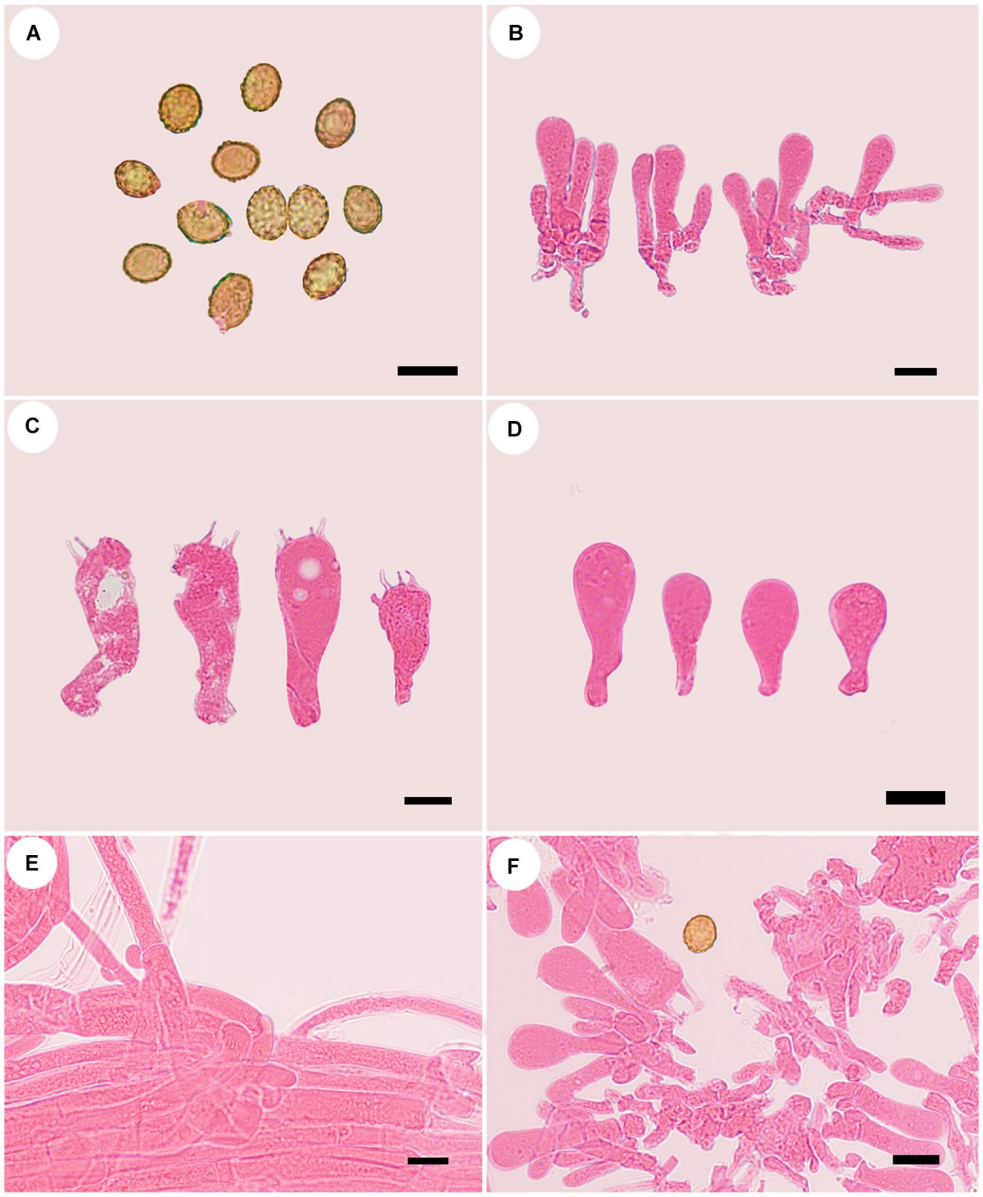
Microscopic structures of *Cortinarius vinoso-griseum* (holotype, FLF 463). **(A)** Basid-iospores; **(B)** Basidioles; **(C)** Basidia; **(D)** Basidioles; **(E)** Hyphae; **(F)** A section of the hymenium. Scales 10 μm.

***Cortinarius vinoso-griseum* B.Y. Wang, T. F. Ma & L.F. Fan sp. nov.** (Figures 6, 7).

**MycoBank No**: 854912.

**Etymology**: Vinoso-griseum (Latin) refers to the violaceous gray pileus.

**Holotype**: CHINA. Gansu Province, Zhuoni County, Dayu Valley, July 2023, FLF 463 (MHGAU).

**Diagnosis**: Differs from *C. ferrugineifolius* by its wider basidiospores.

**Description**: Basidiomes small-to medium-sized. Pileus 1.5–3.8 cm, at first broadly convex, then lower convex to plane, broadly umbonate at the center, margin incurved; at first violaceous gray, tinged cigar brown at the center, finely fibrillose, with brown universal veil remains at the margin; surface silky when dry or glutinous when wet. Context thin, creamy white, soft. Lamellae adnate to adnexed, lilac, moderately distant, sometimes margin wavy. Stipe cylindrical to clavate, gradually slender toward the apex, 3.2–4.9 cm long, 1.9–3.6 cm wide, violaceous buff when young then fading to white tint, leaving an ochraceous ring on the upper stem, hollow. Odor indistinct.

Basidiospores [100/5/5] 7.5–9.7× 5.6–7.8 μm, av. 9.7 × 7.8 μm, Q = 1.24, ellipsoid to broadly ellipsoid to subglobose, yellowish brown, moderately verrucose, without amyloid and dextrinoid reaction. Basidia (15.0–) 25.0–40.0 × (6.2–)7.0–13.6(−14.5) μm, 4-spored, sterigmata up to 2.0–5.0 μm, clavate to subcylindrical, colorless or with granules. Pileipellis duplex, hyphae 7.0–14.0 μm wide, epicutis gelatinous, 58.0–108.0 μm thick, composed of colorless, irregularly arranged and strongly interwoven hyphae, hypocuits 22.0–35.0 μm thick, composed of colorless or amber yellow, nearly parallel cylindrical hyphae. Lamellar edges fertile. Cystidia absent. Lamellar trama uniform, 45.0–60.0 μm thick, composed of parallel arranged hyphae, hyphae 6.0–8.0 μm wide. Stipitipellis gelatinous, stipe hyphae 11.0–13.0 μm wide, thin-walled, cylindrical, interwoven.

**Habitat, ecology and distribution**: Solitary to gregarious on mixed coniferous and broad-leaved forestland, from Gansu, China, July to September. Additional specimens examined. China, Gansu Province: Zhuoni County, Taohe National Nature Reserve, at 34.1175°N, 103.6293°E, alt. 2,800 m, 28 July 2022, L.F. Fan and B.Y. Wang, (WBY463, MHGAU).

**Notes**: *Cortinarius vinoso-griseum* can be differentiated from other species of section *Fusispori* for its violaceous gray pileus, usually distributed under mixed coniferous and broad-leaved forestland at 2,800 m. In addition, basidiospores broadly globose.

**Figure 8:**
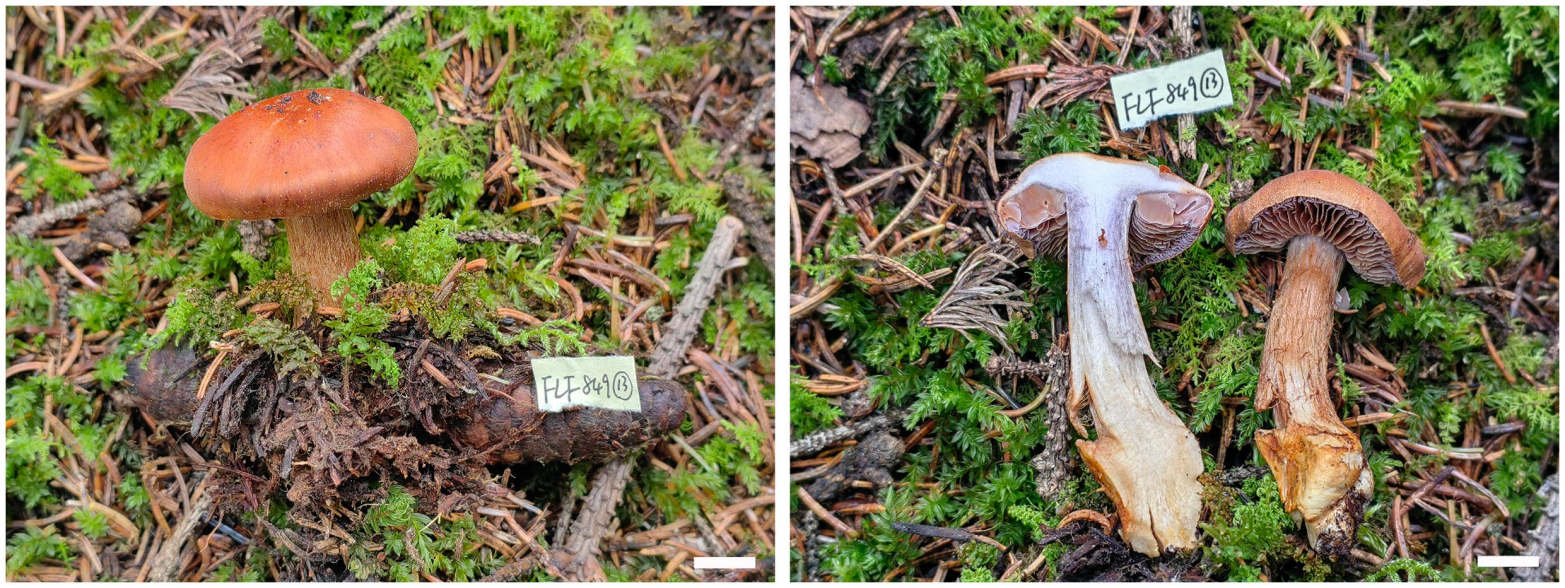
Basidiomes of *Phlegmacium subcalyptratum* (Holotype, FLF 849). Bars: 1 cm.

**Figure 9:**
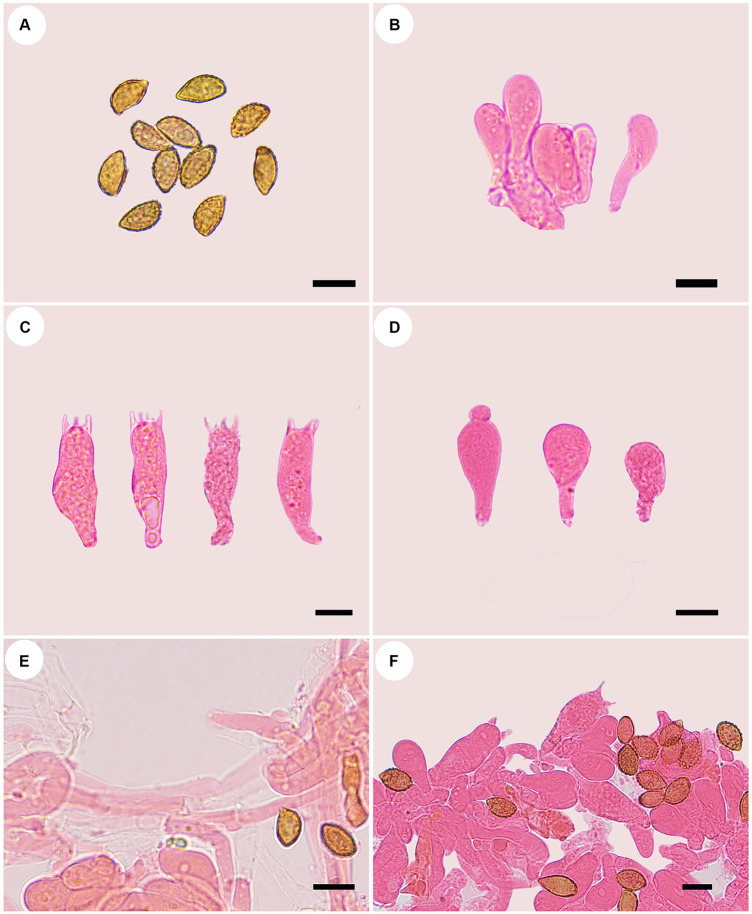
Microscopic structures of *Phlegmacium subcalyptratum* (holotype, FLF 849). **(A)** Ba-sidiospores; **(B)** Basidioles; **(C)** Basidia; **(D)** Cystidia; **(E)** Hyphae with clamp connections; **(F)** A section of the hymenium. Scales 10 μm.

***Phlegmacium subcalyptratum* B.Y. Wang, T.F. Ma & L.F. Fan sp. nov.** (Figures 8, 9).

**MycoBank No**: 854913.

**Etymology**: Subcalyptratum (Latin) refers to the species being similar to *Phlegmacium calyptratum*.

**Holotype**: CHINA. Gansu Province, Zhuoni County, Cheba Valley, September 2023, FLF 849 (MHGAU).

**Diagnosis**: Differs from *P. calyptratum* by its fibrillose pileus.

**Description**: Basidiomes small-to medium-sized. Pileus 3.8–4.0 cm, broadly convex, broadly umbonate at the center, margin incurved or decurved to upturned; at first apricot-orange, tinged coral at the center, smooth, with brown universal veil remains at the margin; surface silky when dry or glutinous when wet. Context thin, white, soft, lavender when bruised. Lamellae adnexed, fawn, moderately distant, sometimes margin wavy. Stipe cylindrical to clavate, slightly bent, gradually slender toward the apex, up to 6.0 cm long, 1.8 cm wide, cinnamon when young then fading to saffron tint, leaving an ochraceous ring on the upper stem, hollow. Odor indistinct.

Basidiospores [100/5/5] (9.7–) 10.0–12.7 (−13.6) × (4.6–) 5.6–6.8 (−7.3) μm, av. 11.3 × 6.2 μm, Qm = 1.82, fusiform, rarely subglobose, yellowish brown, moderately verrucose, without amyloid and dextrinoid reaction. Basidia (25.9–) 29.0–36.0 × 8.0–10.6(−11.5) μm, four-spored, sterigmata up to 2.6–5.0 μm, clavate to subcylindrical, colorless or with amber yellow oily inclusions or granules. Pileipellis duplex, hyphae 8.0–14.0 μm wide, with clamp connections, epicutis strongly gelatinous, 60.0–85.0 μm thick, composed of colorless or amber yellow, irregularly arranged and strongly interwoven hyphae, hypocuits 25.0–40.0 μm thick, composed of colorless or amber yellow, nearly parallel cylindrical hyphae. Lamellar edges fertile. Cystidia clavate, 14.3–19.4 × 9.6–11.7 μm. Lamellar trama regular, 45.0–80.0 μm thick, composed of parallel arranged hyphae, hyphae 5.0–7.0 μm wide. Stipitipellis gelatinous, stipe hyphae 5.0–11.0 μm wide, thin-walled, cylindrical, interwoven.

**Habitat, ecology and distribution**: Usually solitary on coniferous forest land, from Gansu, China, July to September. Additional specimens examined. China, Gansu Province: Zhuoni County, Taohe National Nature Reserve, at 34.737533 °N, 103.485595 °E, alt. 2,870 m, 31 July 2022, L.F. Fan and B.Y. Wang, (WBY 849, MHGAU).

**Notes**: *Phlegmacium subcalyptratum* can be differentiated from other species for its apricot-orange pileus, usually under coniferous forest land at 2,870 m. In addition, basidiospores fusiform.

## 5 Discussion

The main aim of this study was to carry out a molecular revision of *Cortinarius* s. l. species from Northwestern China and to define characteristics useful for delimiting and redefining species. The phylogenetic analysis showed 12 sections, sect. *Delibuti*, *Phlegmacium*, *Anomali*, *Spilomei*, *Bolares*, *Camphorati*, *Dermocybe*, *Leprocybe*, *Subtorti*, *Liyuorum*, *Orellani*, and *Fusispori*, with strong statistical support. However, the phylogenetic position of *Cortinarius fusisporus* and other taxa is still unclear as no supported sister relationship was revealed in the phylogenetic analysis, and the new clade, *Fusispori* clade, was proposed.

*Cortinarius flammeouraceus* and *C. subargyronotus* resemble *C. gansuensis* by having brown pilei, but *C. flammeouraceus* is different from *C. gansuensis* by having shorter basidiospores (7.5–8.5 μm vs. 8.5–10.6 μm, Niskanen, 2020); *C. subargyronotus* is different from *C. gansuensis* by having ellipsoid basidiospores (Liimatainen, 2014). *Cortinarius evernius* (Fr.) Fr. resembles *C. gansuensis* by having brown pilei and long stipe, but the latter has a white stipe and broader pilei (Malloch et al., 2024).

*Cortinarius tricholomoidus* is related to *C. cotoneus*, but *C. cotoneus* is different from *C. tricholomoidus* due to its olivaceus pileus (Liimatainen et al., 2022). *Cortinarius tricholomoidus* is similar to *C. caninoide* by having small-to-medium brown basidiomes with a bulbous stipe, but the latter has smaller basidiospores (6.1–7.5 × 3.8–4.7 μm vs. 7.4–8.5 × 6.2–7.3 μm, Malloch et al., 2024). *Cortinarius hemitrichus* resembles *Cortinarius tricholomoidus* by having a felty pileus, but the former has narrower basidiospores (4.2–5.8 μm vs. 6.2–7.3 μm, Xie, 2018).

According to our phylogenetic analysis, *Cortinarius vinoso-griseum* sisters to *C. ferrugineifolius*, *C. fusisporus*, and *C. umbrinolens*. However, *C. ferrugineifolius* is different from *C. vinoso-griseum* by having a violaceous gray pileus and shorter basidiospores (7.3–11.3 × 4.3–6.3 μm vs. 7.5–9.7 × 5.6–7.8 μm, Moser, 1993); *C. fusisporus* is different from *C. vinoso-griseum* by having larger basidiospores (9.5–11.5 μm vs. 7.5–9.7 μm, Kühner, 1955); *C. umbrinolens* has narrower basidiospores (5.6–7.8 vs. 4.3–4.8 μm, Xie, 2022). *Cortinarius vinoso-griseum* is easily confused with *C. pseudobiformis* in dark brown basidiomes, but the latter has strongly dextrinoid basidiospores (Malloch et al., 2024).

*Phlegmacium subcalyptratum* resembles *Cortinarius armillatus* and *Cortinarius gentilis* (Fr.) Fr. by having an orange pileus, but the latter has a bigger pileus (5–11 cm vs. 3.8–4 cm, Fries, 1838) and shorter basidiospores (7.8–9.7 μm vs. 10.0–12.7 μm).

Northwestern China boasts of the most significant virgin forests in the country, which serve as vital habitats for a variety of unique macrofungi. These forests create ideal environments for these specialized fungi to thrive in and play a crucial role in maintaining the delicate balance of the ecosystem (Xie, 2018; Zhou et al., 2022; Xie et al., 2022).

## Data Availability

The datasets presented in this study can be found in online repositories. The names of the repository/repositories and accession number(s) can be found in the article/Supplementary material.
