# Supplementary material for: Four new species of Cortinariaceae (Agaricales) from Northwestern China
Source: Front Microbiol. 2024 Sep 25;15:1454736. doi: 10.3389/fmicb.2024.1454736 (PMC11461355; doi:10.3389/fmicb.2024.1454736)
Supplement: Supplementary file 1 [file Table_1.DOCX]

| **Species** | **Sample no.** | **Country** | **GenBank** | |
| --- | --- | --- | --- | --- |
|  |  |  | ITS | LSU |
| *Cortinarius anomalus* | CFP1154 typus | Sweden | KX302224 |  |
| *C. anomalus* | CA3 | Norway | KC842425 | KC842495 |
| *C. anomalus* | TUB011883 | Europe, Germany | AY669645 | AY669645 |
| *C. barlowensis* | JFA13140 | North America | FJ717554 |  |
| *C. bolaris* | T40 | Europe, Norway | KC842426 | KC842496 |
| *C. bolaris* | TUB0118524 | Germany | AY669596 | AY669596 |
| *C. bolaris* | 3861 | Canada | KJ705110 |  |
| *C. bolaris* | CFP1008 typus | Sweden | KX302233 |  |
| *C. calaisopus* | PDD103678 | New Zealand | KF727395 | KF727338 |
| *C. calaisopus* | PDD94050 | Dunedin,  New Zealand | NR157880 | MH108373 |
| *C. camphoratus* | SMI193 | Canada,  North America | FJ039626 |  |
| *C. camphoratus* | TRTC175623 | Canada | PP383785 |  |
| *C. caninus* | HMJAU44372 | China | OP620657 | OP620671 |
| *C. caninus* | CFP627 typus | Sweden | KX302250 |  |
| *C.cinnamomeus* | UBCF19609 | Canada | HQ604650 | HQ604650 |
| *C.cinnamomeus* | OS480 | Norway | KC842413 | KC842483 |
| *C. cotoneus* | 19XML11153 | China | OP620655 | OP620666 |
| *C. cotoneus* | OS579 | Norway | KC842423 | KC842493 |
| *C. cruentoides* | PDD101864 typus | New Zealand | KJ635217 | KJ635217 |
| *C. cruentoides* | JAC13529 | New Zealand | MW263695 | MW263408 |
| *C. delibutus* | F17048 | Canada,  North America | FJ717515 |  |
| *C. delibutus* | OS574 | USA,  North America | KC842441 | KC842511 |
| *C. dysodes* | PDD70499 typus | New Zealand | GU233340 | GU233394 |
| *C. dysodes* | PDD72664 | New Zealand | MH101614 | MH108334 |
| *C. epsomiensis* | KM74963 typus | United Kingdom | MK010952 |  |
| *C. epsomiensis* | HMJAU44505 | China | ON254423 |  |
| *C. ferrugineifolius* | IBMMoser19910305 | Europe,  North America | NR171327 |  |
| *C. ferrugineifolius* | SHLindstromCFP969 | Europe,  North America | MT935278 |  |
| *C. ferrusinus* | JB810613 | Spain | KY657254 |  |
| *C. ferrusinus* | JB888116 | Spain | KY657255 |  |
| *C. fibrillososalor* | MHHNU32070 | East Asia,  China, Hunan | OR660685 | OR647503 |
| *C. fibrillososalor* | MHHNU32494 | East Asia,  China, Hunan | OR647481 | OR647506 |
| *C. flammeouraceus* | H6029919 | Europe,  North America | NR170035 |  |
| *C. flammeouraceus* | HMJAU60648 | China | OL891470 |  |
| *C. fusisporus* | BILAS51600 | Lithuania | ON406294 |  |
| *C. fusisporus* | BILAS51540 | Lithuania | ON261481 |  |
| ***C. gansuensis*** | **FLF814** | **China** | **PP911501** | **PP907035** |
| ***C. gansuensis*** | **WBY814** | **China** | **PP911502** | **PP907036** |
| *C. illibatus* | HMJAU48760 | China | MW911735 | OP620668 |
| *C. illibatus* | iNat13972929 | USA | OK346478 |  |
| *C. indotatus* | PDD88257 | New Zealand | KJ421110 | KJ421110 |
| *C. indotatus* | PDD92040 | New Zealand | GU222322 |  |
| *C. liyui* | HMJAU58939 typus | Jilin, China | OP620660 | OP620672 |
| *C. liyui* | HMJAU58938 | Jilin, China | OP620661 |  |
| *C. luhmannii* | TUB019811 | Germany | KJ421114 |  |
| *C. luhmannii* | TUB019808 | Germany | KJ421111 |  |
| *C. pseudocamphoratus* | HMJAU48698 holotype | China | OM001483 | OM001524 |
| *C. pseudocamphoratus* | HMJAU48798 | China | OM001489 |  |
| *C. pseudosalor* | MHHNU8349 | East Asia,  China, Hunan | OR647352 |  |
| *C. pseudosalor* | MHHNU32148 | East Asia,  China, Hubiei | OR660688 | OR647505 |
| *C. pseudosalor* | MHHNU32082 | East Asia,  China, Hubiei | OR660686 | OR647504 |
| *C. putorius* | TNO7411HT | USA,  North America | KR011124 |  |
| *C. rotundisporus* | PDD96298 | New Zealand | MH101550 | MH108389 |
| *C. rotundisporus* | PDD72611 | Australia,  New Zealand | AY669612 | AY669612 |
| *C. salor* | TUB011838 | Europe, Germany | AY669592 | AY669592 |
| *C. sommerfeltii* | HMJAU44457 | China | OP620652 | OP620663 |
| *C. sommerfeltii* | SOMF30854 | Spain | OQ398585 |  |
| *C. sp.* | SWUBC741 | Canada | DQ481671 |  |
| *C. sp.* | T21468 | China | OP620656 | OP620667 |
| *C. sp.* | MEL2089705 | Australia | GQ890326 | JX544951 |
| *C. spilomeus* | TUB011523 | Europe | AY669654 | AY669654 |
| *C. spilomeus* | CFP1137 typus | Sweden | KX302267 |  |
| *C. spilomeus* | H6031514 | Finland | KX302264 |  |
| *C. subargyronotus* | H7018127 | Finland | NR131871 |  |
| *C. subargyronotus* | C358 | Hungary | OP099768 |  |
| *C. subsalor* | HMJAU48759 typus | China | MW911734 | OP620670 |
| *C. subsalor* | HMJAU48758 | China | MW911733 |  |
| *C. subsanguineus* | HMJAU48961 | China | OP620653 | OP620664 |
| *C. subsanguineus* | HMAS250503 | China | MK411450 |  |
| *C. subtortus* | F16111 | North America | FJ157044 | FJ157044 |
| *C. subtortus* | TUB011382 | Europe | AY174857 | AY174857 |
| *C. subtropicus* | MHHNU31981 | East Asia,  China, Hunan | OR660687 | OR647502 |
| *C. subtropicus* | MHHNU33533 | East Asia,  China, Hunan | OR647488 | OR647508 |
| *C. tabularis* | CFP949 typus | Sweden | KX302275 |  |
| *C. tabularis* | H7022440 | Finland | KX302279 |  |
| *C. tasmacamphoratus* | HOA20606A0 | Tasmania | AY669633 | AY669633 |
| *C. tessiae* | PDD107517 | New Zealand | MG019356 | MG019356 |
| *C. tetonensis* | JFA10350 | North America | MZ580436 |  |
| *C. tibeticisalor* | HMJAU48764 typus | China | MW911729 | OP620669 |
| *C. tibeticisalor* | HMJAU48763 | China | MW911730 |  |
| ***C. tricholomoidus*** | **FLF806** | **China** | **PP911497** | **PP907031** |
| ***C. tricholomoidus*** | **FLF827** | **China** | **PP911498** | **PP907032** |
| *C. uliginosus* | KH7 | Norway | KC842412 | KC842482 |
| *C. uliginosus* | TUB011823 | Germany | AY669584 | KJ403804 |
| *C. umbrinolens* | TUB011918 | Germany | AY669658 |  |
| *C. umbrinolens* | NFSG20231021 | Britain | PP355760 |  |
| *C. veronicae* | PDD68468 typus | New Zealand | KC017355 |  |
| *C. veronicae* | JAC10781 | New Zealand | MW263653 | MW263361 |
| ***C. vinoso-griseum*** | **FLF463** | **China** | **PP911499** | **PP907033** |
| ***C. vinoso-griseum*** | **WBY463** | **China** | **PP911500** | **PP907034** |
| *C. viridipileatus* | OTA61977 | New Zealand | MK546592 | MK546595 |
| *C. viridipileatus* | OTA64087 | New Zealand | MK546593 | MK546596 |
| *C. xiaojinensis* | HMJAU58895 | China | OP620654 | OP620665 |
| *C. xiaojinensis* | HMAS274355 | China | MK411447 |  |
| *C. saginus* | T30 | Norway | KC842448 | KC842518 |
| *C. saginus* | IB19960705 | USA | AF325608 | AF388768 |
| *Phlegmacium caerulescens* | Fungal | Turkish | MH718791 | MH718792 |
| *P. caerulescens* | SF44815 | Frisia | NR130199 |  |
| *P. calyptratus* | iNAT18441433 | America | OL602058 |  |
| *P. calyptratum* | MICH10328 | Frisia | NR130201 |  |
| *P. glaucocephalus* | IB19950679 | Frisia | NR130221 |  |
| *P. glaucocephalus* | HBAU15487 | China | MW862302 |  |
| *P. neotriumphans* | G2970631 | Frisia | NR157947 |  |
| *P. neotriumphans* | HMAS260251 | China | OK490097 |  |
| *P. populinum* | OF58605 | Norway | MT216235 |  |
| *P. populinum* | O58647 | Europe,  Australia,  Tasmania,  New Zealand,  South America | AY669521 |  |
| ***P. subcalyptratum*** | **FLF849** | **China** | **PP911503** | **PP907037** |
| ***P. subcalyptratum*** | **WBY849** | **China** | **PP911504** | **PP907038** |
| *C. eartoxicus*  (outgroup) | MEL2351137 | Australia | KP311432 | KP311376 |
| *C. eartoxicus*  (outgroup) | MEL2151441 | Australia | OK159884 |  |
| *C. orellanus*  (outgroup) | IB19980580 | Austria | AF389164 | AF388773 |
| *C. rubellus*  (outgroup) | TUB011828 | Germany | AY669595 | AY669595 |
